# Prostate Cancer Presenting With Isolated Mediastinal Metastasis: A Case Report

**DOI:** 10.7759/cureus.107460

**Published:** 2026-04-21

**Authors:** Andrew Sawan, Tiffany Chen, Cameron Bondy, Kambiz Raoufi

**Affiliations:** 1 Internal Medicine, California University of Science and Medicine, Colton, USA; 2 Internal Medicine, Arrowhead Regional Medical Center, Colton, USA

**Keywords:** atypical metastasis, lymph node metastasis, mediastinal lymphadenopathy, mediastinal metastasis, metastatic prostate cancer, prostate adenocarcinoma, prostate-specific antigen

## Abstract

Prostate cancer typically metastasizes in a predictable pattern, most commonly to the axial skeleton through Batson's venous plexus with associated regional pelvic lymphadenopathy. Mediastinal lymph node involvement is rare and represents an atypical route of dissemination that may lead to diagnostic uncertainty, particularly when thoracic findings predominate. We present a case of a 56-year-old Hispanic male with no significant past medical history with biopsy-confirmed prostatic adenocarcinoma with hematogenous mediastinal spread. CT imaging revealed extensive mediastinal, retroperitoneal, and pelvic lymphadenopathy with prostatic enlargement. This case highlights mediastinal lymph node metastasis as an uncommon initial manifestation of advanced prostate cancer and underscores the importance of tissue diagnosis in patients presenting with atypical thoracic adenopathy. Recognition of unusual metastatic patterns improves the understanding of tumor biology and may prevent misdiagnosis.

## Introduction

Prostate cancer is one of the most common malignancies among men worldwide, with an estimated 1.5 million new cases and 397,000 deaths globally in 2022, making it the second most frequently diagnosed cancer and the fifth leading cause of cancer death among men [1]. Advanced disease most frequently involves the axial skeleton, producing osteoblastic lesions via hematogenous dissemination through Batson’s venous plexus, along with regional pelvic lymph node involvement [2]. Visceral metastases may occur in late-stage disease; however, mediastinal lymph node involvement is uncommon and not part of the typical lymphatic drainage pathway of the prostate.

The lymphatic drainage of the prostate comprises three major routes: ascending lymphatics leading to the external iliac nodes, lateral ducts leading to the hypogastric and internal iliac nodes, and posterior lymphatics directed to the pararectal and presacral lymph nodes [3]. Supradiaphragmatic lymph nodes, including mediastinal and supraclavicular nodes, represent distant metastatic sites and are not included in the regional pelvic lymphatic drainage basin according to current staging classifications [4].

Atypical patterns of metastasis are clinically significant because they may obscure the primary diagnosis, particularly when thoracic lymphadenopathy precedes genitourinary symptoms. In such cases, the differential diagnosis often includes lymphoma or primary pulmonary malignancy. Reporting rare metastatic presentations contributes to improved understanding of tumor biology, alternative lymphatic pathways, and mechanisms of disease progression. Additionally, documentation of unusual dissemination patterns adds to the literature regarding staging implications and prognosis in advanced prostate cancer. We present a case of metastatic prostate adenocarcinoma initially manifesting with mediastinal and supraclavicular lymphadenopathy, highlighting the diagnostic challenges and clinical implications of this atypical presentation.

## Case presentation

A 56-year-old Hispanic male with no significant past medical history except for cholecystitis status post cholecystectomy, as well as prior tobacco and cocaine use, presented with a progressive cough that began two months prior to admission. Following the onset of cough, he developed left supraclavicular lymphadenopathy. While awaiting an outpatient biopsy, he experienced a one-month history of worsening shortness of breath, hoarseness of voice, and mild dysphagia to solids, prompting evaluation in the emergency department.

On physical examination, he was noted to have hoarseness and palpable left supraclavicular lymphadenopathy. Laboratory evaluation revealed a prostate-specific antigen level of 806 ng/mL, aspartate aminotransferase of 69 U/L, and lactate dehydrogenase of 1096 U/L. Hepatitis C antibody testing was reactive, with a hepatitis C viral RNA quantitative real-time polymerase chain reaction (HCV RNA, QN, real-time PCR) of 7.03 log IU/mL.

**Figure 1:**
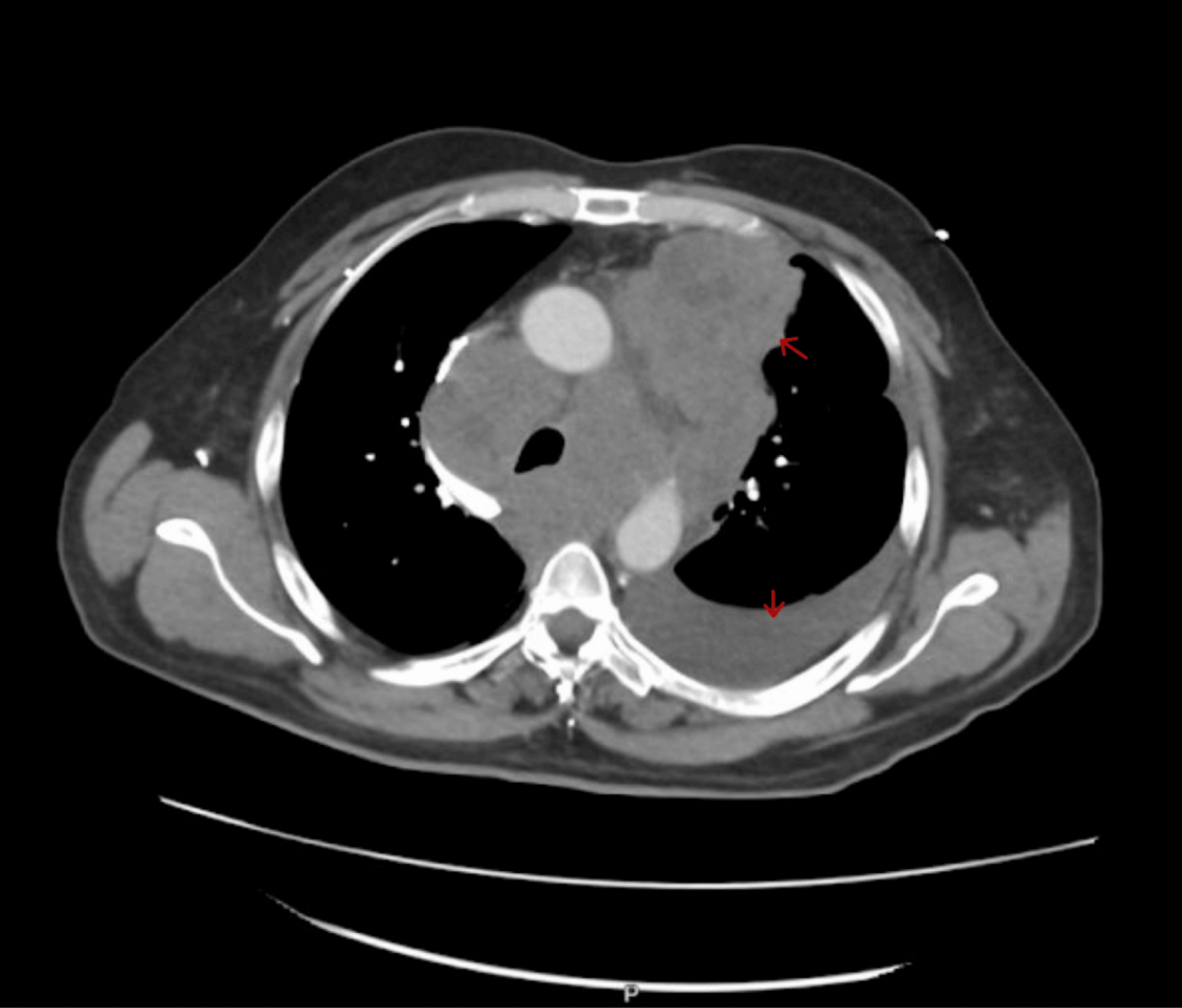
CT chest with contrast axial view shows mediastinal mass with left-sided pleural effusion and extensive mediastinal adenopathy

Computed tomography (CT) of the chest with contrast revealed extensive mediastinal adenopathy with large lymph nodes surrounding the aorta, trachea, esophagus, brachiocephalic vessels, and extension to the supraclavicular region (Figure 1).

**Figure 2:**
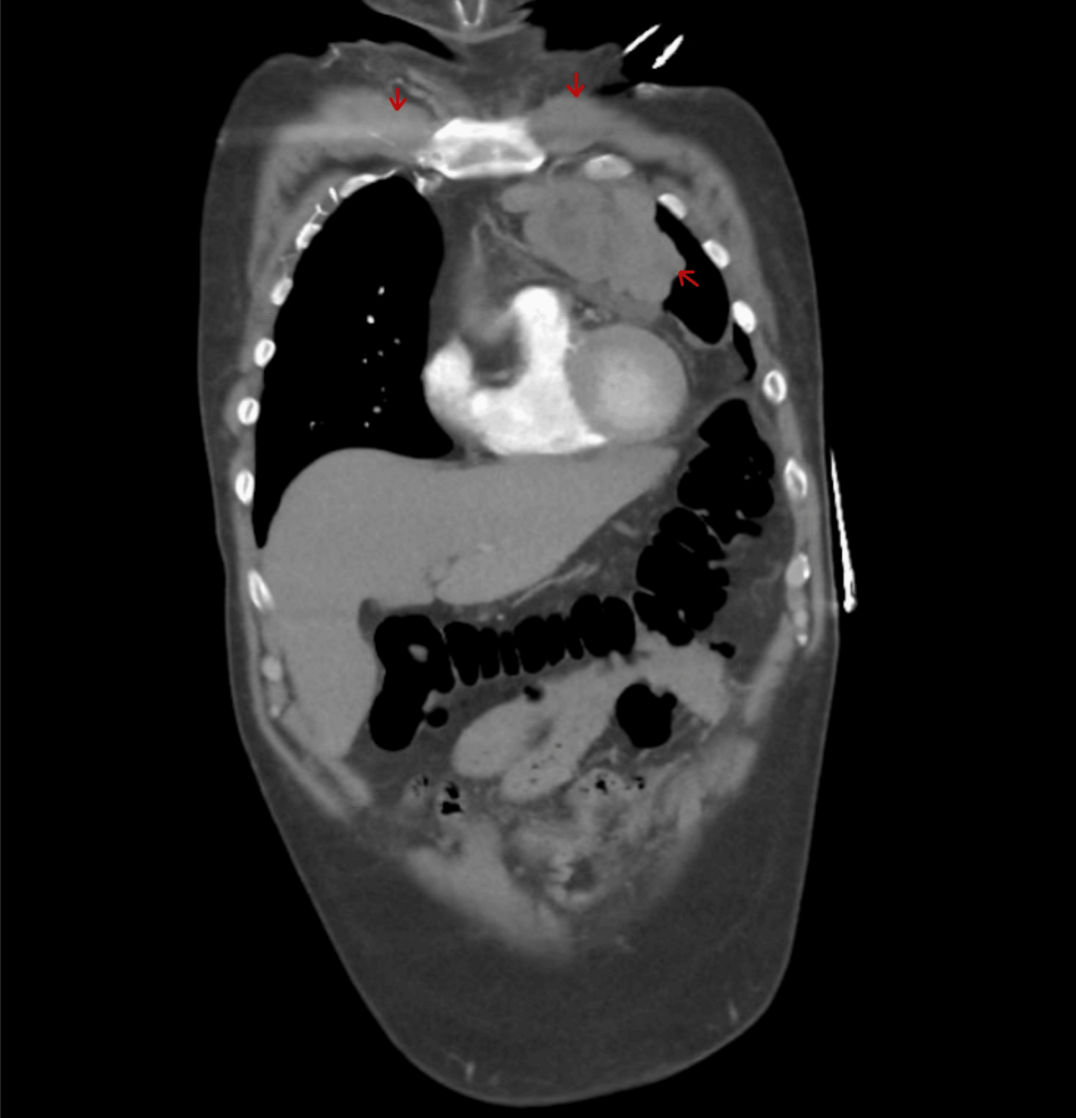
CT chest coronal view shows a mediastinal mass and extensive bilateral supraclavicular lymphadenopathy

CT abdomen with contrast demonstrated significant retroperitoneal adenopathy with lymph nodes surrounding the aorta and inferior vena cava measuring up to 4 cm (Figure 2).

**Figure 3:**
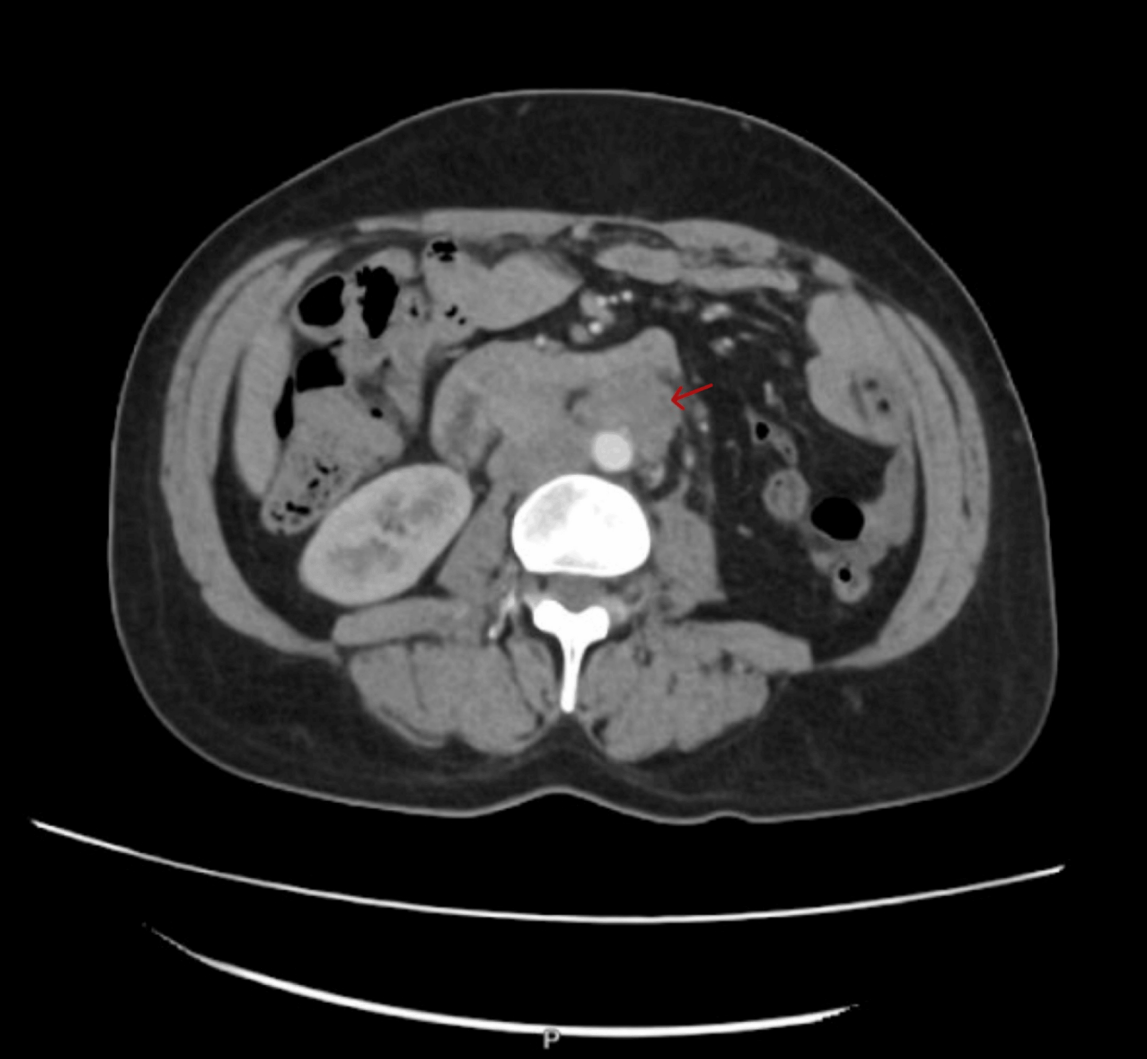
CT abdomen and pelvis axial view shows an enlarged right internal iliac lymph node

**Figure 4:**
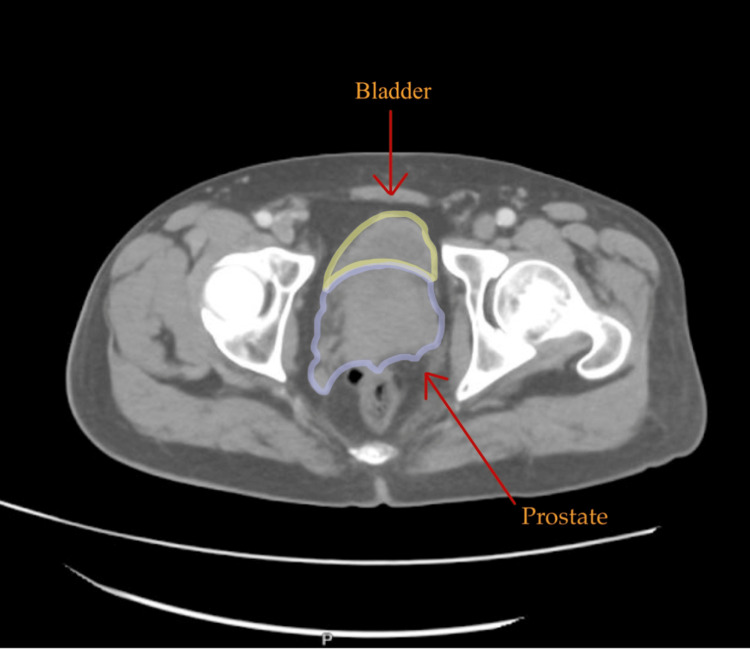
CT abdomen pelvis axial view demonstrates an enlarged prostate The bladder was not well-distended for evaluation.

CT pelvis with contrast showed a large right internal iliac lymph node measuring up to 3 cm, prostatic enlargement, and intact osseous structures (Figures 3-4).

The combined laboratory and Imaging findings supported the initial concern for metastatic malignancy. Excisional biopsy of the left supraclavicular lymph node revealed lymphoid tissue infiltrated by high-grade, poorly differentiated carcinoma characterized by increased nuclear-to-cytoplasmic ratios, irregular hyperchromatic nuclei, prominent nucleoli, and scant cytoplasm, consistent with metastatic adenocarcinoma. Immunohistochemical staining demonstrated positivity for pancytokeratin, alpha-methylacyl-coenzyme A racemase (AMACR), NK3 homeobox 1 (NKX3.1), and MoC31, and negativity for cytokeratin 7, caudal-type homeobox 2, napsin A, p40, and thyroid transcription factor 1. These findings supported a prostatic primary source of metastasis.

Oncology was consulted and recommended initiating bicalutamide while inpatient and leuprolide four weeks later. Radiation oncology recommended a nuclear bone scan, which, unfortunately, must be completed on an outpatient basis, at which point, the patient will be transferred to another oncology group due to insurance incompatibility.

## Discussion

Prostate cancer characteristically metastasizes in a predictable pattern, most commonly to the axial skeleton through Batson’s venous plexus with associated regional pelvic lymphadenopathy. Autopsy studies of 1,589 patients have demonstrated that bone metastases occur in approximately 90% of patients with hematogenous spread, with a gradual decrease in spinal involvement from the lumbar (97%) to cervical (38%) levels, consistent with retrograde venous dissemination through the vertebral venous plexus followed by upward spread along spinal veins [5]. Mediastinal lymph node involvement represents a deviation from this established pathway and suggests dissemination beyond the conventional lymphatic drainage of the prostate. The mechanism underlying this atypical spread is not fully understood but may involve retrograde lymphatic flow, hematogenous dissemination bypassing the vertebral venous plexus, or aggressive tumor biology with altered metastatic tropism. Extensive nodal disease outside the expected drainage basin may reflect advanced or high-grade pathology capable of exploiting alternative routes of spread.

In this case, thoracic symptoms and supraclavicular lymphadenopathy preceded recognition of a prostatic primary, raising initial concern for primary pulmonary or lymphoproliferative malignancy. Definitive diagnosis required histopathologic confirmation. Immunohistochemical positivity for α-methylacyl-CoA racemase (AMACR) and NK3 homeobox 1 (NKX3.1), with absence of pulmonary and gastrointestinal markers, established prostatic origin. These findings highlight the importance of comprehensive tissue analysis when evaluating metastatic adenocarcinoma of unclear primary, particularly when clinical presentation is atypical. Corroborating clinical findings, including elevated prostate-specific antigen levels and prostate imaging, further supported the diagnosis of metastatic prostate adenocarcinoma.

AMACR is consistently upregulated in prostate cancer and demonstrates high sensitivity for prostate carcinoma compared with benign prostate tissue [6]. NKX3.1 has emerged as a highly sensitive and specific marker for metastatic prostatic adenocarcinoma and performs particularly well in poorly differentiated tumors where traditional markers such as PSA or prostatic acid phosphatase may be expressed at low levels or not at all [7]. The combination of AMACR positivity with NKX3.1 nuclear staining, in the absence of pulmonary markers such as thyroid transcription factor-1 (TTF-1) and napsin A or gastrointestinal markers such as CDX2, provides strong immunohistochemical evidence for prostatic origin in metastatic adenocarcinoma of unknown primary [6,7].

Although mediastinal metastasis from prostate cancer has been reported, it remains uncommon relative to the frequency of osseous and pelvic nodal involvement. Earlier clinical series examining metastatic patterns of prostate cancer have demonstrated that atypical metastatic sites, including lung, liver, and supradiaphragmatic lymph nodes, occur far less frequently than bone metastases and generally reflect advanced systemic disease [8]. Historical case series have documented that metastatic prostate carcinoma to supradiaphragmatic lymph nodes occurs predominantly on the left side, with reported involvement of supraclavicular, cervical, axillary, and mediastinal nodes. Notably, these reports demonstrate that prostate cancer may occasionally present with supraclavicular lymphadenopathy in the absence of classic osseous metastases or abnormal rectal examination findings [9]. Several additional case reports have described prostate cancer presenting as an isolated supraclavicular mass, pleural effusion, or mediastinal lymphadenopathy, with immunohistochemical staining for prostatic markers proving essential for establishing the diagnosis [10,11].

Supraclavicular lymphadenopathy, particularly on the left side, is highly suggestive of malignancy across age groups, with approximately 54-84% of cases attributable to malignancy originating from thoracic or abdominopelvic organs [12]. Left supraclavicular lymphadenopathy has long been recognized as a potential manifestation of abdominal or pelvic malignancy due to lymphatic drainage through the thoracic duct into the left supraclavicular nodal basin [13]. In the American Joint Committee on Cancer (AJCC) eighth edition staging system, supraclavicular lymph node involvement is classified as distant lymph node metastasis (M1a disease) rather than regional nodal disease [4].

Current National Comprehensive Cancer Network (NCCN) guidelines recommend androgen deprivation therapy (ADT) combined with androgen receptor pathway inhibitors (ARPIs), such as abiraterone, apalutamide, or enzalutamide, as preferred first-line therapy for metastatic castration-sensitive prostate cancer [14]. Large randomized clinical trials have demonstrated that the addition of ARPIs to ADT significantly improves overall survival compared with ADT alone [15,16]. For patients with supradiaphragmatic nodal involvement representing distant metastatic disease, systemic therapy with ADT plus ARPI is therefore strongly recommended, with consideration of metastasis-directed therapy in selected patients with oligometastatic disease [14,17].

Recognition of atypical thoracic dissemination may prevent diagnostic delay and misclassification as a primary thoracic malignancy. Furthermore, documentation of such cases contributes to a broader understanding of metastatic behavior and disease progression in advanced prostate cancer. Contemporary imaging with prostate-specific membrane antigen positron emission tomography (PSMA-PET) has further enhanced detection of atypical metastatic sites and may reveal patterns of dissemination not previously appreciated with conventional imaging modalities [18,19].

## Conclusions

This case illustrates an uncommon but clinically significant presentation of metastatic prostate adenocarcinoma manifesting primarily with mediastinal and supraclavicular lymphadenopathy in the absence of osseous involvement. The initial thoracic symptoms and supradiaphragmatic lymphadenopathy raised concern for primary pulmonary malignancy or lymphoproliferative disease, underscoring the diagnostic challenge posed by atypical metastatic patterns. Immunohistochemical analysis with prostatic markers, particularly NKX3.1 and AMACR, proved essential for establishing the diagnosis. Clinicians should maintain a high index of suspicion for prostatic origin when evaluating metastatic adenocarcinoma of unknown primary, especially in men with elevated PSA levels, even when the presentation is atypical. This case contributes to the growing literature documenting atypical metastatic patterns in prostate cancer and reinforces the value of tissue diagnosis and comprehensive immunohistochemical profiling in patients presenting with unexplained thoracic lymphadenopathy.
